# Predicting surgical outcome in intractable epilepsy using a computational model of seizure initiation

**DOI:** 10.1186/1471-2202-16-S1-P230

**Published:** 2015-12-18

**Authors:** Nishant Sinha, Justin Dauwels, Yujiang Wang, Sydney S Cash, Peter N Taylor

**Affiliations:** 1 School of Electrical and Electronics Engineering, Nanyang Technological University, Singapore; 2 School of Computing Science, Newcastle University, Newcastle upon Tyne, UK; 3 Massachusetts General Hospital and Harvard Medical School, Boston, MA, USA

## 

A third of patients with epilepsy are refractory to anti-epileptic drug treatment. For some of these patients with focal epilepsy, better seizure control can be achieved by surgical treatment in which the seizure focus is localized and resected while avoiding crucial cortical tissues. However, approximately 30% of the patients continue to have seizures even after surgery. In other words, reliable criteria for patient's outcome prediction are absent. Computational models with appropriate parameter setting and patients specific connectivity allows an exciting opportunity to make predictions based on the model dynamics.

In this study, non-seizure (inter-ictal) epoch of electrographic recording has been used to calculate the functional synchrony between different cortical regions. This synchrony measure was then used as the connectivity parameter in a computational model of transitions to a seizure like state. Hypothesizing that the network synchrony plays an important role in determining the likelihood of surgical success, we retrospectively analyzed 19 patients having intractable epilepsy, who underwent surgical treatment to achieve seizure freedom. All data were collected confirming to ethical guidelines and under protocols monitored by the local Institutional Review Boards according to NIH guidelines.

Building upon the computational model in [1], the regions which were more likely to transit into a seizure like state were delineated. It was found that these regions are correlated with those identified by clinicians as the seizure onset zone. Moreover, it was found that the resection of these regions in the model reduces the overall likelihood of a seizure. The likelihood of a surgical success was calculated *in silico *by iteratively increasing the area of resection and the surgical outcomes were successfully predicted for 14 out of 19 patients.

The methods presented here may aid clinicians to delineate the seizure focus. Moreover, it may facilitate neurosurgeons in predicting the likelihood of a surgical success and to investigate alternative cortical tissues to operate on if the seizure focus is in the eloquent cortex.

**Table 1:** Prediction of surgical outcomes

**No**.	Age group at onset	Age group at surgery	Surgical Resection	Outcome (Engel Class)	Predicted Outcome
1	21-30	21-30	Right Temporal lobe	Seizure Free (II)	Good outcome

2	41-50	41-50	Right Temporal lobe	Seizure Free (I)	Good outcome

3	21-30	21-30	Left Cingulate	Seizure Free (I)	Bad outcome

4	41-50	41-50	Left Temporal	Seizure Free (I)	Good outcome

5	11-20	11-20	Right Parietal	Seizure Free (I)	Good outcome

6	51-60	51-60	Amygdalohippocampectomy Left Medial Frontal Lobe	Seizure Free (I)	Bad outcome

7	11-20	11-20	Right anterior-superior frontocortical Right Temporal Lobe, Amygdalohippocampectomy	Seizure Free (I)	Good outcome

8	11-20	11-20	Left occipital brain lobe	Seizure Free (I)	Bad outcome

9	31-40	31-40	Right frontal lobe	Seizure Free (I)	Good outcome

10	1-10	1-10	Left lateral frontal cortex, Left anterior frontal cortex Mesial left frontal cortex	Seizure Free (I)	Bad outcome

11	21-30	21-30	Left FrontoTemporal	Not Seizure Free	Bad outcome

12	31-40	31-40	Right Temporo-Occipital Region	Not Seizure Free (IV)	Bad outcome

13	21-30	21-30	Right Temporal Lobe	Not Seizure Free (IV)	Bad outcome

14	11-20	11-20	Left Anterior Temporal Lobe Amygdalohippocampectomy	Not Seizure Free (V)	Good outcome

15	1-10	1-10	Left Parietal Lobe	Not Seizure Free (IV)	Bad outcome

16	1-10	1-10	Right Frontal Lobe	Not Seizure Free (IV)	Bad outcome

17	31-40	31-40	Left Temporal	Not Seizure Free (V)	Bad outcome

18	21-30	21-30	Left Temporal Lobe	Not Seizure Free (V)	Bad outcome

19	1-10	1-10	Left Frontal Lesion	Not Seizure Free (V)	Bad outcome
